# Effect of Gelatin-Stabilized Copper Nanoparticles on Catalytic Reduction of Methylene Blue

**DOI:** 10.1186/s11671-016-1656-6

**Published:** 2016-10-01

**Authors:** Aminu Musa, Mansor B. Ahmad, Mohd Zobir Hussein, Mohd Izham Saiman, Hannatu Abubakar Sani

**Affiliations:** 1 Department of Chemistry, Faculty of Science, Universiti Putra Malaysia, 43400 UPM, Serdang, Selangor Malaysia; 2 Advanced Materials and Nanotechnology Laboratory, Institute of Advanced Technology (ITMA), Universiti Putra Malaysia, 43400 UPM, Serdang, Selangor Malaysia; 3 Department of Pure and Industrial Chemistry, Faculty of Natural and Applied Sciences, Umaru Musa Yar’adua University, Katsina, Dutsin-ma Road, P.M.B 2218, Katsina, 820001 Nigeria

**Keywords:** Gelatin, Catalytic reduction, Methylene blue, Copper nanoparticles

## Abstract

The synthesis of copper nanoparticles was carried out with gelatin as a stabilizer by reducing CuSO_4_.5H_2_O ions using hydrazine. Ascorbic acid and aqueous NaOH were also used as an antioxidant and pH controller, respectively. The effects of NaOH, hydrazine, and concentration of gelatin as stabilizer were studied. The synthesized copper nanoparticles were characterized by UV-vis spectroscopy, XRD, zeta potential measurements, FTIR, EDX, FESEM, and TEM. The formation of CuNPs@Gelatin is initially confirmed by UV-vis spectroscopic analysis with the characteristic band at 583 nm. XRD and TEM reports revealed that CuNPs@Gelatin (0.75 wt.%) is highly crystalline and spherical in shape with optimum average size of 4.21 ± 0.95 nm. FTIR studies indicated the presence of amide group on the surface of the CuNPs indicating the stability of CuNPs which is further supported by zeta potential measurements with the negative optimum value of −37.90 ± 0.6 mV. The CuNPs@G4 showed a good catalytic activity against methylene blue (MB) reduction using NaBH_4_ as a reducing agent in an aqueous solution. The best enhanced properties of CuNPs@G4 were found for the 0.75 wt.% gelatin concentration. Thermodynamic parameters (Δ*H* and Δ*S*) indicate that under the studied temperature, the reduction of MB by CuNPs@G4 is not feasible and had endothermic in nature.

## Background

Copper nanoparticles are a less expensive other option to different precious metal nanoparticles with a range of prospective users in the area of nanoscience and technology [1]. However, the preparation of CuNPs has been broadly concentrated on for a long time as it is an essential industrial material because of its unique physicochemical properties. Likewise, in the area of electronics, copper is the most widely recognized as a result of its excellent electrical conductivity and additionally low cost [2]. Similarly, CuNPs have engrossed great attention in catalytic applications. In any case, CuNPs have significant impediments, which incorporate quick oxidation on subjection to atmospheric air. Copper oxidizes to Cu_2_O and CuO and convert to Cu^2+^ during preparation and storage, so it is hard to prepare CuNPs without an inert environment [3]. Along these lines, another method has been developed to prepare CuNPs in the presence of polymer and surfactants as stabilizers and to form covers on the surface of nanoparticles. These stabilizers are mostly from non-renewable materials, finding eco-friendly stabilizing material is needed.

Gelatin is an animal protein obtained by a controlled hydrolysis of the fibrous insoluble collagen present in the bones and the skin produced as waste during animal slaughtering and processing [4]. It possesses an important properties, such as flexibility, adhesiveness, and low cost, which make it suitable for practical application in various fields of research [5]. Similarly, gelatin contains free carboxyl groups on its backbone and has the potential for chelating and reducing noble metals. Few works on the preparation of gelatin-stabilized CuNPs have been reported [6, 7]. However, the preparation of CuNPs has become a subject of interest in material research, several synthesis methods of CuNPs with controlled size and shape have been reported, including sonochemical reduction [8, 9], laser ablation [10], microemulsion [11], thermal deposition [12], chemical reduction [13], microwave [14], and green method [15]. Among the methods, chemical reduction is the most extensively applied methods for its simplicity, low cost, and ease of size and shape control over CuNPs. CuNPs have also been reported as a suitable catalyst for chemical reduction of various organic pollutants in wastewater [16–18]. Besides, many researchers have reported the reduction of dyes using various metal nanoparticles [19–27]. However, the use of CuNPs for the reduction of aromatic dyes has remained an unexplored area.

However, to the best of our knowledge, no work on CuNPs@Gelatin catalyst for the reduction of methylene blue dye has been reported. In an effort to develop a green and cost-effective catalyst to address the said environmental issue, in this work, we report a simple method for the preparation of CuNPs stabilized with gelatin, using copper sulfate, NaOH solution, and hydrazine hydrate and ascorbic acid as copper precursor, pH controller, reducing agent, and to prevent the oxidation of CuNPs, respectively, without any inert atmosphere at a temperature of 80 °C. The effect of gelatin concentrations on the catalytic activity of CuNPs against the chemical reduction of methylene blue using NaBH_4_ as a hydrogen donor was studied. Likewise, the thermodynamic parameters for reduction reaction has been looked into.

## Methods

### Materials

CuSO_4._5H_2_O (99 %) was used as copper ions precursor and was provided by Bendosen Laboratory Chemicals, ascorbic acid (90 %) was provided by Hamburg, NaOH (99 %) and hydrazine hydrate (35 % hydrazine) were purchased from MERCK (Germany), and gelatin (type B), ethanol, methylene blue and NaBH_4_ (98.5 %) were purchased from Sigma-Aldrich (USA). In this, all the preparation of solutions, chemical of analytical reagent grade, and deionized water were used.

### Synthesis of Copper Nanoparticles in Gelatin

For the synthesis of copper nanoparticles (CuNPs) in gelatin, five different gelatin suspensions were first prepared by dissolving 0.5, 0.38, 0.25, 0.13, and 0.05 g of the gelatin in five different flasks containing 50 mL warm distilled water each at 40 °C to achieve 1, 0.75, 0.5, 0.25, and 0.1 % (*w*/*v*) suspensions. After that, 15 mL of CuSO_4_.5H_2_O (0.1 M) were added to 35 mL each of 1, 0.75, 0.5, 0.25, and 0.1 % (*w*/*v*) of gelatin suspensions to get the final concentration of 0.03 M. Then, 2.5 mL of 0.02 M ascorbic acid was added with constant stirring at 80 °C for 20 min. This was followed by the addition of 5 ml of NaOH solution, after further mixing for another 20 min, until a light green solution was obtained. Finally, 2.5 mL of 35 wt.% hydrazine was added for reduction of copper ions during which the solution mixture change from dark to reddish brown within 30 min of the reaction time with constant stirring. The CuNPs@Gelatin was secluded by centrifugation at 14,000 rpm for 10 min and dried in a vacuum overnight at 60 °C.

### Catalytic Activity of CuNPs@Gelatin

An investigation of the catalytic activity of the prepared copper nanoparticles supported in different percentages of gelatin was carried out according to a previous work by [28], using the reduction of methylene blue dye by NaBH_4_ as a model reaction. Briefly, 10 mg of the prepared samples were added to 18 mL of methylene blue aqueous solution (1 × 10^−5^ M). Subsequently, the above solution was mixed with 2 mL fresh NaBH_4_ solution (1 × 10^−2^ M). The reaction was done in the given temperature with continuous stirring. The progress of the degradation reaction was then monitored by recording the absorbance value at 664 nm at different time interval using UV-vis spectrophotometer. The concentration of the methylene blue was calculated based on a calibration curve of the absorbance values versus dye concentrations. Also, blank experiments were carried out to show that the reactions do not proceed without catalysts only in the presence of NaBH_4_. The influence of the temperature and recyclability of the optimum samples were studied.

### Characterization of the Synthesized CuNPs@Gelatin

The initial characterization was carried out by UV-vis spectroscopic study using a UV 1650 PC-Shimazu B UV-visible spectrophotometer (Shimazu, Osaka, Japan). The XRD analysis of the prepared CuNPs@Gelatin was carried out by Philip X’pert PXRD (Cu K*α* radiation; PANalytical, Almedo, The Netherlands). The prepared CuNPs@Gelatin was also subjected to zeta potential measurements using a dynamic laser light scattering method in a Malvern zeta instrument 3000 (Malvern Instrument, UK). FTIR spectra of the samples were obtained at ambient temperature using the KBr disk method. A disk containing 1 mg of sample was recorded within the wavenumber range of 200 to 4000 cm^−1^ using a series 100 Perkin Elmer (USA) FT-IR 1650 spectrophotometer. The components of the samples were measured by the energy dispersive x-ray spectroscopy (EDX). The morphology and size of the prepared CuNPs@Gelatin were examined using FESEM and TEM. The FESEM with EDX analysis was performed with a JEOL JSM-7600F instrument. The transmission electron microscopy (TEM) observation was carried out using TEM, Philips CM-12, and the particle size distribution was measured using UTHSCSA Image Tool version 3.0. Also, the histograms were created using IBM-SPSS software, and the graph fitting was created using Microsoft Excel program.

## Results and Discussion

The mechanism of formation for CuNPs in a gelatin solution as the stabilizer using the chemical reduction method using hydrazine is proposed by the following equations.

1 $$ {\mathrm{Cu}}^{2+}+\mathrm{Gelatin}\to {\left[\left(\mathrm{Gelatin}/\mathrm{C}\mathrm{u}\right)\right]}^{2+} $$

2 $$ {\left[\left(\mathrm{Gelatin}\right)/\mathrm{C}\mathrm{u}\right]}^{2+}+2{\mathrm{OH}}^{-}\to {\left[\left(\mathrm{Gelatin}\right./\mathrm{C}\mathrm{u}{\left(\mathrm{O}\mathrm{H}\right)}_2\right]}^{2+} $$

3 $$ {\left[\left(\mathrm{Gelatin}/\mathrm{C}\mathrm{u}\right.{\left(\mathrm{O}\mathrm{H}\right)}_2\right]}^{2+}+{\mathrm{N}}_2{\mathrm{H}}_{4\left(\mathrm{a}\mathrm{q}\right)}\ \to \left[\mathrm{Gelatin}/\mathrm{Cu}\mathrm{NPs}\right]+{\mathrm{N}}_2+2{\mathrm{H}}_2\mathrm{O}+{\mathrm{H}}_2 $$

**Fig. 1:**
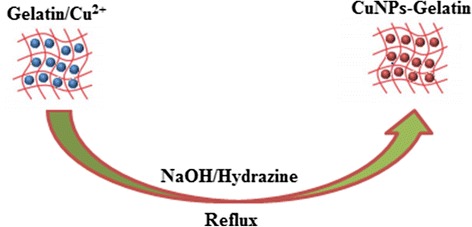
Schematic illustration for the formation mechanism of CuNPs in gelatin using N_2_H_4_ as reducing agents

A schematic illustration for the formation mechanism of CuNPs in gelatin using N_2_H_4_ as reducing agents is proposed in Fig. 1.

**Fig. 2:**
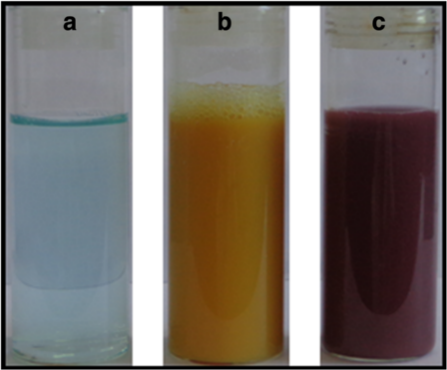
Photograph of CuNPs@Gelatin at various concentrations of gelatin [0.1, 0.25, 0.5, 0.75, and 1 wt.%, respectively (**a**–**c**)], respectively

The color changes from light blue to yellow upon addition of NaOH solution, and after addition of hydrazine, color changes to red wine as shown in the Fig. 2a–c, respectively.

### Optimization of Synthesis Method for the Preparation of CuNPs@Gelatin

To prepare a stable small size CuNPs, different parameters such as concentrations of gelatin, concentrations of CuSO_4_, volume of NaOH, and volume of hydrazine were investigated. The optimum values were found to be 0.75 wt.% of gelatin, 0.03 M concentration of Cu^2+^ ions, 4 mL of 0.5 M NaOH, and 2 mL of 35 wt.% hydrazine concentration, respectively. These values were required to obtain a stable small CuNPs in an aqueous solution.

### UV-Visible Spectroscopy

**Fig. 3:**
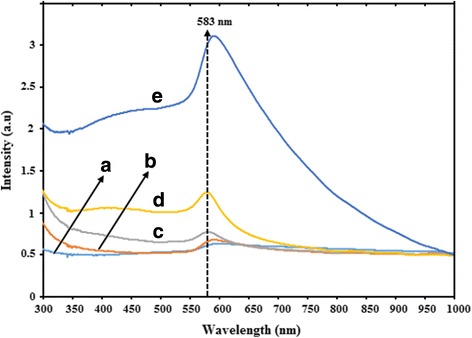
UV-vis absorption spectra of CuNPs@Gelatin with various concentrations of gelatin [0.1, 0.25, 0.5, 0.75, and 1 wt.% (*a*–*e*)]

UV-vis absorbance spectroscopy has ended up being very useful methods for studying metal nanoparticles in light of the fact that the peak positions and shapes are sensitive to particle size. The effect of gelatin concentrations on the UV-vis absorbance spectroscopy of the prepared CuNPs is shown in Fig. 3a–e. The UV-vis spectral profile generated for gelatin-stabilized CuNPs revealed the formation of CuNPs@Gelatin with the maximum wavelength around 583 nm. Figure 3a–e shows that when the concentration of gelatin increased from 0.1 to 1 wt.% (a–e), respectively, the intensity of the SPR peak position also gradually increased. The increase of the absorbance was a characteristic that the concentration of CuNPs increased. Besides, an increase in the concentration of gelatin, the absorbance also increased, which resulted in a blue shift in SPR position from 600 to 592 nm as shown in Fig. 3a, b, which alluded to a decrease in the particle size. Similarly, a red shift in SPR from 583 to 590 nm, which leads to a gradual increase in the CuNPs size.

### X-ray Diffraction

**Fig. 4:**
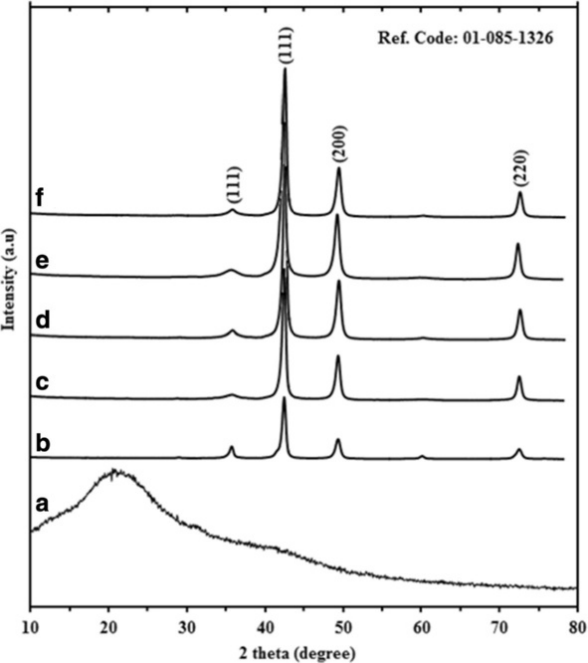
X-ray diffraction patterns of gelatin (*a*) and CuNPs@Gelatin at various concentrations of gelatin [0.1, 0.25, 0.5, 0.75, and 1 wt.% (*b*–*f*)], respectively

The formation and stability of CuNPs@Gelatin were confirmed through XRD phase analysis. A typical XRD patterns obtained for the CuNPs prepared using a gelatin solution has been shown in Fig. 4a–f. The presence of intense peaks at 36.64°, 43.31°, 50.54°, and 74.15° of the spectrum related to the (111), (111), (200), and (220) individually, indexed a crystalline copper face-centered cubic (FCC) phase [29, 30]. The broad diffraction peak at 22.35° is ascribed to gelatin as shown in Fig. 4a. Similarly, as seen in spectra (b–e) in Fig. 4, the intensity increased with the increasing gelatin concentration, but in Fig. 4f, the intensity reduced due to the high concentration of gelatin. The crystalline sizes of the CuNPs were calculated by means of an X-ray line-broadening method using the Scherrer equation:

4 $$ D=K\lambda /\beta \operatorname{Cos}\theta $$

where *K* is the Scherrer constant with the value from 0.9 to 1 (shape factor), *λ* is the x-ray wavelength (1.54.18Ǻ), *β*_1/2_ is the width of the XRD peak at half height, and *θ* is the Bragg angle. The (111) plane at 43.31° was chosen to calculate the crystalline size (either plane can be used for this purpose). The crystalline size of the CuNPs prepared at different gelatin concentrations of 0.1, 0.25, 0.5, 0.75, and 1 wt.%, respectively, were found to be 26, 22, 19, 16, and 20 nm, respectively.

### Zeta Potential

**Fig. 5:**
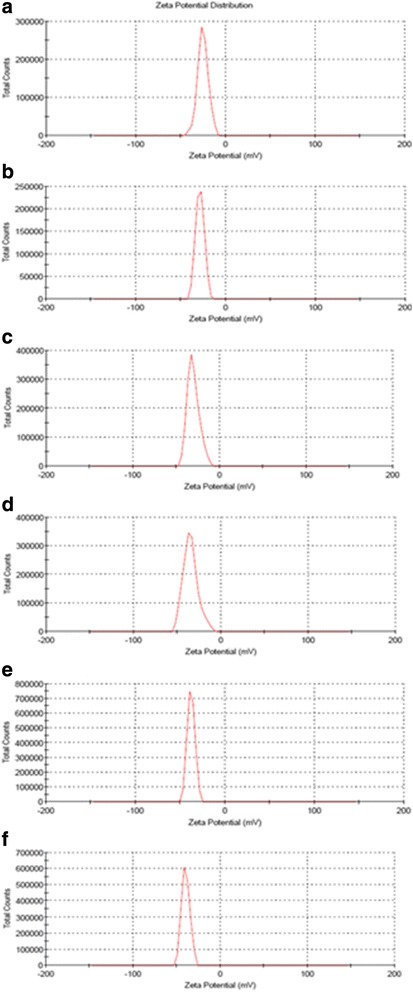
Zeta potential of gelatin (**a**) and CuNPs@Gelatin at various concentrations of gelatin [0.1, 0.25, 0.5, 0.75, and 1 wt.% (**b**–**f**)], respectively

The determination of zeta potential was carried out to understand the charges as well as the nanoparticle stability. Figure 5a–f shows the zeta potential of gelatin and CuNPs at various concentrations of gelatin [0.1, 0.25, 0.5, 0.75, and 1 wt.% (b–f)], respectively, in neutral water. All the samples demonstrated a negative zeta potential in aqueous solution, in which gelatin demonstrated the most minimal negative value of −25.4 ± 2.0 mV, and this value was observed to be increased relative with increasing of the concentrations of gelatin as shown in Table 1. The negative potential values developed due to attachment of carboxylic and amino groups on the surface of the CuNPs. Zeta potential is a significant parameter known to affect the stability of colloidal dispersions. Generally, particles with more positive and or positive values than ±30 mV for zeta potential are considered to form stable dispersion [31].

**Table 1:** Zeta potential of gelatin and CuNPs@Gelatin at various concentrations of gelatin [0.1, 0.25, 0.5, 0.75, and 1 wt.% (b–f)]

Samples	Zeta potential (mV)
a	−25.4 ± 2.0
b	−28.7 ± 1.4
c	−30.4 ± 0.8
d	−34.8 ± 1.1
e	−37.9 ± 0.6
f	−39.7 ± 0.9

### Fourier Transform Infrared Spectroscopy

**Fig. 6:**
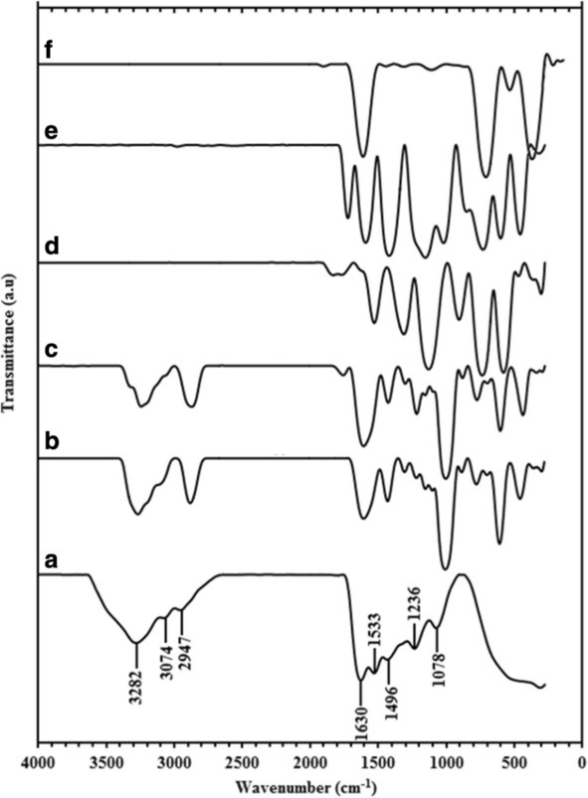
FTIR spectra for gelatin (*a*) and CuNPs@Gelatin at various concentrations of gelatin, [0.1, 0.25, 0.5, 0.75, and 1 wt.% (*b*–*f*)], respectively

The possible interaction between gelatin and CuNPs was studied using FTIR as shown in the Fig. 6b–f. The FTIR spectrum of gelatin shown in Fig. 6a exhibits characteristic peaks similar to that reported in previous works [32, 33]. The main absorption bands for the gelatin are identified with various vibration methods of the peptide bond, which include amide A, amide I, amide II, and amide III. Amide A is connected with the N–H stretching vibration of hydrogen-bonded amide groups at 3600–2300 cm^−1^. Notwithstanding to amide A band, in the same district, the O–H stretching vibration of water molecules appeared. Amide I is related to C=O stretching vibration at 1630 cm^−1^. Amide II is connected to C–N stretching vibration and N–H bending vibration at 1533 cm^−1^. Finally, amide III is related to C–N stretching vibration at 1236 cm^−1^ [34, 35]. The spectra of CuNPs@Gelatin show no change in amide I, which showed that there were no real changes in secondary structure of gelatin because of the interaction between the gelatin molecule and CuNPs. The formation of CuNPs made the peaks of H–bond (3282 cm^−1^) and amide band (1533 cm^−1^) to be shifted slightly to the left as observed in Fig. 6b, c, and the amide bands at (1533 cm^−1^) completely disappeared as shown in Fig. 6d–f, respectively. These proposed that there is electrostatic crosslinking between the CuNPs and the gelatin, in this manner affirming the capping of the CuNPs by gelatin.

### Energy Dispersive X-ray Spectroscopy

**Fig. 7:**
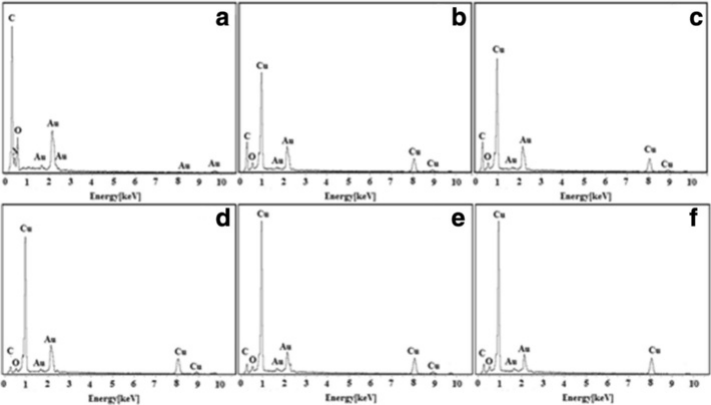
EDX spectroscopy for gelatin and CuNPs@Gelatin at various concentrations of gelatin [0.1, 0.25, 0.5, 0.75, and 1 wt.% (**a**–**f**)], respectively

The chemical compositions of gelatin and prepared CuNPs@Gelatin were analyzed by EDX. Figure 7a–f shows EDX patterns of gelatin and CuNPs@Gelatin at various concentrations of gelatin (0.1, 0.25, 0.5, 0.75, and 1 wt.%), respectively. The signals for elemental copper in the EDX spectra in Fig. 7b–f demonstrated that CuNPs were stabilized by gelatin. The presence of carbon and oxygen in the EDX spectra was attributed to gelatin. The peaks at 1.75 to 2.25 keV are identified to gold, which were utilized for sample coating. The height of Cu peaks and the amount of NPs increased with the increasing gelatin concentration.

### Microscopic Analysis

**Fig. 8:**
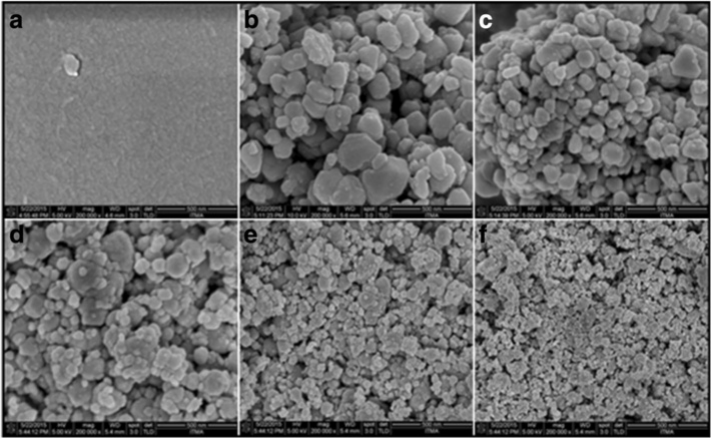
FESEM micrographs for gelatin and CuNPs@Gelatin at various concentrations of gelatin [0.1, 0.25, 0.5, 0.75, and 1 wt.% (**a**–**f**)], respectively

**Fig. 9:**
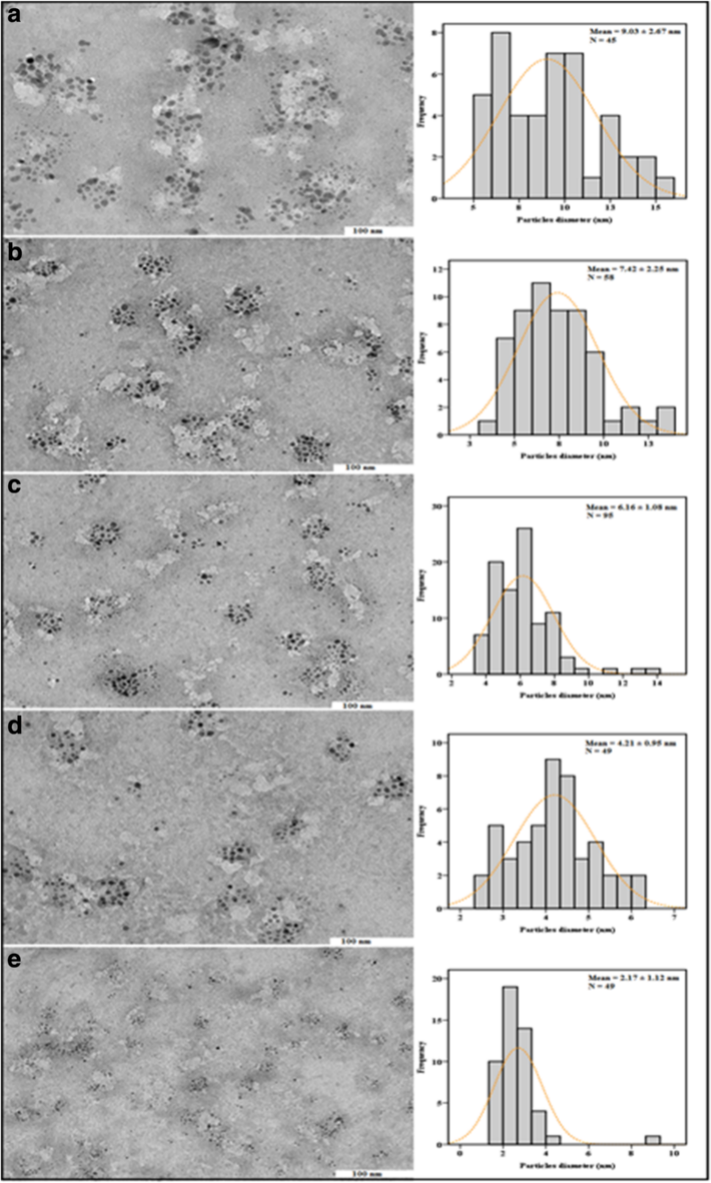
TEM images and histograms showing particles size distribution for CuNPs@Gelatin at various concentrations of gelatin [0.1, 0.25, 0.5, 0.75, and 1 wt.% (**a**–**e**)], respectively

The FESEM images of gelatin and CuNPs at different gelatin concentration of (0.1, 0.25, 0.5, 0.75, and 1 wt.%) are exhibited in Fig. 8a–f, respectively. The micrographs obtained for the various gelatin concentrations demonstrate that the CuNPs were supported within the gelatin matrix. The particles have different sizes and distribution patterns based on the gelatin concentrations. Figure 9a–e displays TEM micrographs and their corresponding particle size distributions of CuNPs at various concentrations of gelatin. TEM images and their size distributions indicated that the mean diameters and standard deviation of CuNPs were found to be 9.03 ± 2.67, 7.42 ± 2.25, 6.16 ± 1.08, 4.21 ± 0.95, and 2.17 ± 1.12 nm for 0.1, 0.25, 0.5, 0.75, and 1 wt.%, respectively. These results revealed that with increasing concentration of the gelatin, the mean diameters of the CuNPs gradually decreased to become smaller particles. This is predictable with the outcomes acquired from different characterizations led on the CuNPs. Moreover, the low standard deviation seen for the 0.75 wt.% gelatin is due to the higher nanoparticle size distribution in the sample as observed in the histogram Fig. 9d. This emphasizes the relevance of gelatin in controlling the size of the nanoparticles, as reported by [36].

### Catalytic Activity of CuNPs@Gelatin

**Fig. 10:**
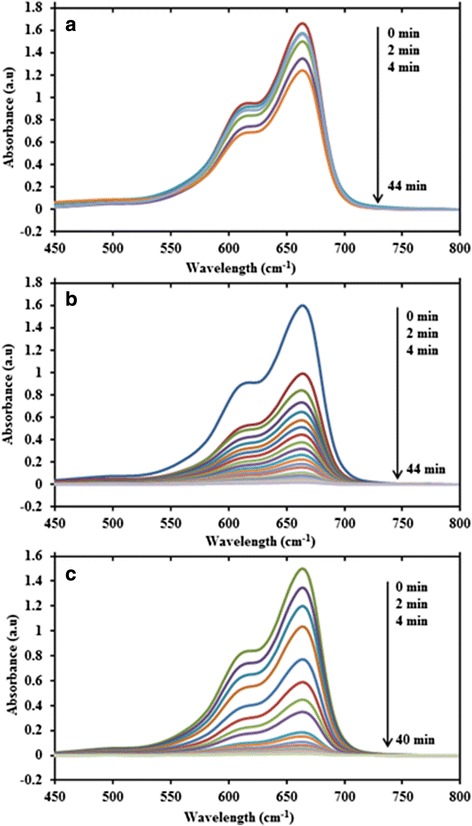
Time dependent UV-vis spectra for the reduction of MB catalyzed with NaBH4 (**a**), CuNPs (**b**) and CuNPs@Gelatin (0.1 wt.%) (**c**)

**Fig. 11:**
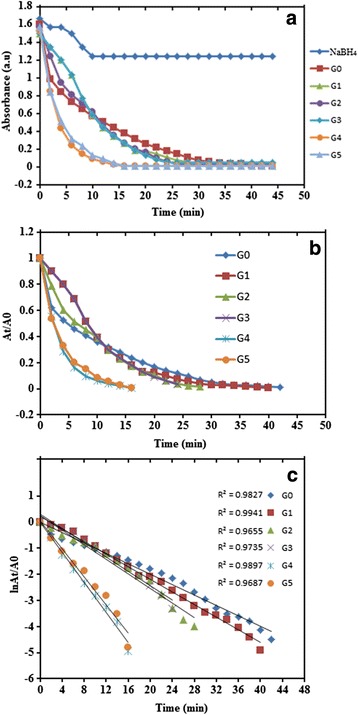
Plot of absorbance (**a**), A_t_/A_0_ (**b**), and ln A_t_/A_0_ (**c**) against time for the MB reduction with NaBH4, CuNPs (N0), and CuNPs@Gelatin at different concentration of gelatin [G1, G2, G3, G4, and G5 (0.1, 0.25, 0.5, 0.75, and 1 wt.%)], respectively

The catalytic activity of the prepared CuNPs@Gelatin for the reduction MB using NaBH_4_ was carried out in the same way reported earlier in this chapter. Figure 10a shows the UV-vis spectra of MB and NaBH_4_ mixture without CuNPs, which it was seen that the reaction is moderate and was not finished after 44 min. Similarly, Fig. 10b, c shows a typical absorption profile of the reduction of MB using CuNPs (G0) and CuNPs@Gelatin (G1), which demonstrates a reduction in the absorption maximum of MB at *λ*_max_ = 664 nm for the complete reduction at a time of 44 and 40 min for (G0) and (G1), respectively. Figure 11a, b demonstrates a reduction in absorbance and A_t_/A_0_ as a function of time of CuNPs and CuNPs@Gelatin at various concentrations of gelatin [0.1, 0.25, 0.5, 0.75, and 1 wt.% (G1, G2, G3, G4, and G5], respectively. Figure 11c shows a good linear correlation of ln (A_t_/A_0_) against time, indicating that the catalytic reduction reaction of MB proceeded with the pseudo-first-order behavior due the use of excess NaBH_4_. Completion time, pseudo-first-order rate constant, and correlation coefficient (*R*^2^) at 25 °C for CuNPs and CuNPs@Gelatin at different concentrations of gelatin, calculated from the slope were shown in the Table 2. The pseudo-first-order rate constant and completion time of the optimum sample of CuNPs@Gelatin (G4) are 0.5748 and 16 min which is almost times compared with that of CuNPs (G0). The catalytic performance of CuNPs@Gelatin was superior to that of CuNPs (G0) as a result of the utilization of gelatin as a capping agent. The result indicated that the reduction time decreases with an increasing gelatin concentration, but there is no time difference at G4 and G5, of which a good linear relationship was obtained for ln (A_t_/A_0_) against time, in G4 which was found to be the optimum sample.

**Table 2:** Completion time and rate constants of CuNPs (G0) and CuNPs@Gelatin at different concentrations of gelatin [G1,G2, G3, G4, and G5 (0.1, 0.25, 0.5, 0.75, and 1 wt.%)] catalyzed reduction reaction of MB

Samples	Completion time (min)	Rate constant (*k*_app_) (min^−1^)	*R* ^2^
G0	44	0.2050	0.9789
G1	40	0.2424	0.9941
G2	28	0.2800	0.9655
G3	24	0.2700	0.9735
G4	16	0.5748	0.9897
G5	16	0.5368	0.9687

### Thermodynamic Parameters of CuNPs@G4

**Fig. 12:**
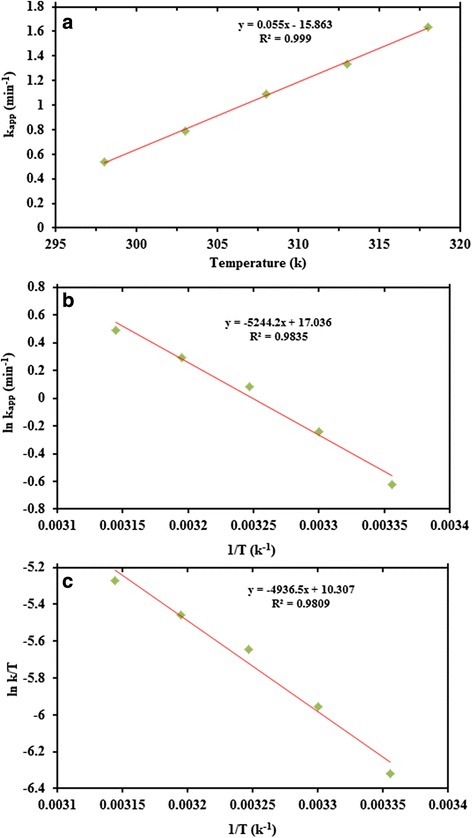
Plot of rate constant from the CuNPs@Gelatin (G4) versus (**a**) temperature, (**b**) Arrhenius plot, and (**c**) Eyring plot for CuNPs@Gelatin (G4)

The rate constant of the catalytic reduction reaction of MB which depends on temperature was evaluated at five different temperatures (25–45 °C), and the resultant apparent rate constants were investigated as appeared in Fig. 12a. Not surprisingly, increasing the temperature brought about an increase in the rate of reaction. From the analyses of various temperature, the activation energy (*E*_*a*_) for the reduction was calculated from the Arrhenius equation (Eq. 5).

5 $$ \ln k= \ln A-\frac{E_a}{RT} $$

The enthalpy change (Δ*H*) and entropy change (Δ*S*) were determined by the Eq. 6.

6 $$ \ln \left(\frac{k}{T}\right)=-\frac{\varDelta H}{RT}+ \ln \frac{kB}{h}+\frac{\varDelta S}{R} $$

The thermodynamic parameters Δ*H* and Δ*S* were determined from (Eq. 6), respectively. Figure 12a shows increase in reaction rate constant as a function of temperature. Figure 12b shows an Arrhenius plot, in which the activation energy was calculated from its slope. However, Fig. 12c shows Eyring plots in which Δ*H* and Δ*S* were determined from its slope and intercept for Δ*H* and Δ*S*, respectively. The values for *E*_*a*_, Δ*H*, Δ*S*, and Δ*G*_298_ for the methylene blue reduction catalyzed by CuNPs@G4 were found to be 43.61 kJ/mol, 41.05 kJ/mol, −111.86 J/mol, and 74.38 kJ/mol, respectively.

## Conclusions

The CuNPs@Gelatin was also successfully prepared in gelatin biopolymer as stabilizer in an aqueous solution, with the variation of the gelatin concentration in the range of 0.1, 0.25, 0.5, 0.75, and 1 wt.%. The prepared CuNPs@Gelatin were initially confirmed by using UV-vis spectroscopy and XRD study. The UV-vis spectral profile generated for gelatin-stabilized CuNPs revealed the formation of CuNPs@Gelatin with a maximum wavelength around 583 nm. According to the XRD and TEM analyses revealed that with increasing concentration of the gelatin, the mean diameters of the CuNPs gradually decrease. The CuNPs@Gelatin showed a good catalytic activity against MB reduction using NaBH_4_ as reducing agent in an aqueous solution. The best enhanced properties of CuNPs@Gelatin were found for the 0.75 wt.% gelatin concentration.
